# Metabolic Dysfunction at the Core: Revisiting the Overlap of Cardiovascular, Renal, Hepatic, and Endocrine Disorders

**DOI:** 10.3390/life16010172

**Published:** 2026-01-20

**Authors:** Maria-Daniela Tanasescu, Andrei-Mihnea Rosu, Alexandru Minca, Andreea-Liana Rosu, Maria-Mihaela Grigorie, Delia Timofte, Dorin Ionescu

**Affiliations:** 1 Department of Semiology-Emergency University Hospital, Carol Davila University of Medicine and Pharmacy, 022328 Bucharest, Romania; maria.tanasescu@umfcd.ro (M.-D.T.);; 2 Department of Cardiology, Prof. Dr. Agrippa Ionescu Emergency Hospital, 077015 Balotesti, Romania; 3 Department of Clinical Pharmacology, BBraun, 013714 Bucharest, Romania; 4 Department of Dentistry, Discipline of Endodontics, Faculty of Dentistry, Carol Davila University of Medicine and Pharmacy, 020021 Bucharest, Romania; 5 Department of Dialysis, Bucharest Emergency University Hospital, 050098 Bucharest, Romania

**Keywords:** metabolic dysfunction, cardiometabolic disease, chronic kidney disease, NAFLD/NASH, endocrine disorders, ectopic fat, inflammation, insulin resistance, metabolic syndrome, multimorbidity, organ crosstalk

## Abstract

Metabolic dysfunction has emerged as a central driver of cardiovascular, renal, hepatic, and endocrine disorders, challenging traditional organ-specific disease models. Increasing evidence indicates that conditions such as obesity, type 2 diabetes, chronic kidney disease, heart failure, and metabolic dysfunction–associated steatotic liver disease frequently develop in parallel, reflecting shared upstream metabolic abnormalities rather than isolated pathologies. This narrative review synthesizes recent clinical, epidemiologic, biomarker, and therapeutic evidence to examine metabolic dysfunction as a unifying framework for multisystem disease, with particular focus on the cardiovascular–renal–hepatic–metabolic (CRHM) model. A targeted literature search of major biomedical databases was conducted to identify relevant studies published between 2020 and 2025, encompassing observational cohorts, randomized trials, and integrative reviews addressing cross-organ metabolic interactions. The reviewed evidence highlights consistent clinical overlap across organ systems, stage-dependent risk amplification and the utility of shared metabolic and inflammatory biomarkers in capturing multisystem vulnerability. In parallel, contemporary metabolic therapies demonstrate coordinated benefits across cardiovascular, renal, and hepatic domains, supporting the concept of common modifiable disease drivers. The reviewed evidence supports a shift from organ-based toward metabolic-centric frameworks for risk stratification and prevention. Viewing metabolic dysfunction as the organizing principle of cardiometabolic disease may improve recognition of multisystem risk, facilitate earlier intervention, and provide a more coherent foundation for precision and preventive medicine, in an era of growing cardiometabolic multimorbidity.

## 1. Introduction

Metabolic dysfunction has emerged as a central, unifying mechanism driving the parallel development of cardiovascular, renal, hepatic, and endocrine disorders, challenging long-standing organ-specific clinical paradigms. Conditions such as obesity, type 2 diabetes, chronic kidney disease (CKD), heart failure, and metabolic dysfunction-associated steatotic liver disease (MASLD) share common upstream drivers, including insulin resistance, adipose tissue dysfunction, ectopic fat deposition, and chronic low-grade inflammation, which promote tightly interconnected disease trajectories rather than isolated pathological entities. Coordinated signaling between the heart, kidneys, liver, vasculature, pancreas, and adipose–muscle axis establishes a metabolic milieu characterized by oxidative stress, inflammatory activation, neurohormonal dysregulation, and impaired substrate utilization, ultimately fostering multisystem injury and clinical multimorbidity [1].

Clinically, the burden of these intersecting disorders is substantial. Metabolic risk factors rarely occur in isolation and instead cluster in epidemiologic patterns consistent with global multimorbidity trends, markedly elevating cardiometabolic risk [2,3]. This clustering reflects a broader recognition that metabolic dysfunction operates as a shared systemic driver across organ systems.

The conceptual understanding of these multisystem interactions has evolved considerably over the past three decades. Early descriptions of Metabolic Syndrome (MetS)—originally framed as “Syndrome X”—highlighted the centrality of insulin resistance and the co-occurrence of obesity, hypertension, and dyslipidemia, with later definitions such as ATP III formalizing its role as a risk factor constellation predisposing individuals to type 2 diabetes and atherosclerotic cardiovascular disease [4]. As evidence accumulated, the impact of metabolic dysfunction on renal physiology became increasingly apparent, leading to the development of the Cardio-Renal Syndrome (CRS) classification, which described acute and chronic bidirectional interactions between the heart and kidneys [5]. Building on this foundation, the American Heart Association proposed the Cardiovascular–Kidney–Metabolic (CKM) syndrome to integrate metabolic risk factors, CKD, and cardiovascular disease within a unified framework. Subsequent observations emphasized the importance of hepatic involvement, particularly MASLD and its progression to metabolic dysfunction-associated steatohepatitis (MASH) and cirrhosis, in amplifying systemic metabolic injury and contributing to both cardiac and renal dysfunction. These insights motivated the development of the Cardio-Renal–Hepatic (CRH) model, acknowledging the liver as a key determinant of multisystem disease trajectories [6].

In 2025, Theodorakis and Nikolaou extended this framework to the Cardiovascular–Renal–Hepatic–Metabolic (CRHM) syndrome, accentuating shared upstream drivers such as metabolic inflammation, insulin resistance, ectopic fat accumulation, and maladaptive adipose–organ signaling as core mechanisms underlying parallel multiorgan dysfunction [7]. This evolution reflects a shift from siloed disease paradigms toward integrated systems-level models that more accurately describe the pathobiology of modern cardiometabolic disease.

Upstream of these multisystem manifestations lies dysfunctional adipose tissue biology. Impaired adipocyte expandability, ectopic lipid overflow, maladaptive adipokine and hepatokine signaling, and chronic inflammatory activation link obesity to hepatic steatosis, insulin resistance, type 2 diabetes, CKD, and cardiovascular disease. Ectopic fat deposition within the liver, myocardium, pancreas, and renal sinus further propagates metabolic toxicity, providing a unifying substrate for the pathophysiology underlying these interrelated disorders [8,9].

Building on the CRHM syndrome proposed by Theodorakis and Nikolaou [7], this narrative review extends the framework by integrating recent human clinical, epidemiologic, biomarker, and therapeutic evidence across organ systems. While the original CRHM model defines shared mechanisms and staging, the present synthesis focuses on how metabolic dysfunction manifests as parallel, stage-dependent cardiovascular, renal, hepatic, and endocrine disease trajectories, and how these pathways are captured by cross-organ biomarkers and modified by contemporary metabolic therapies. This approach operationalizes CRHM as a clinically actionable, phenotype-based model for multisystem risk stratification and precision prevention.

## 2. Materials and Methods

This narrative review was conducted to synthesize current perspectives on the unifying role of metabolic dysfunction across cardiovascular, renal, hepatic, and endocrine disorders, with emphasis on shared biological pathways, clinical overlap, and implications for precision and preventive medicine. A targeted literature search was performed using PubMed, Scopus, Web of Science, and Google Scholar, complemented by manual screening of reference lists to ensure that influential or recently published studies not captured through database queries were included. The search was completed in November 2025 and covered literature published between January 2020 and January 2025, reflecting the period in which multisystem metabolic frameworks have progressively emerged.

The search strategy used Boolean combinations of terms relevant to the conceptual domains of interest, including: “metabolic dysfunction”, “insulin resistance”, “ectopic fat”, “adipose dysfunction”, “cardiometabolic multimorbidity”, “CKM syndrome”, “CRHM framework”, “MASLD”, “NAFLD”, “cardio-renal axis”, “hepato-metabolic dysfunction”, “endocrine-metabolic crosstalk”, and “multisystem inflammation.” These terms were selected to capture mechanisms, clinical intersections, and organ-to-organ communication relevant to the pathophysiology of metabolic dysfunction.

Two reviewers independently screened titles and abstracts for relevance, followed by full-text evaluation of potentially eligible studies. Discrepancies in study selection were resolved through discussion and consensus. Although this review follows a narrative rather than systematic design, methodological transparency was enhanced by incorporating selected elements of systematic review frameworks. Because the goal of this synthesis is conceptual integration across heterogeneous evidence types, including mechanistic studies, clinical reviews, imaging-based analyses, and multi-organ disease models, formal risk of bias tools and quantitative meta-analytic methods were not applied. Instead, emphasis was placed on recency of evidence, conceptual relevance, and translational importance for multisystem metabolic medicine.

Included studies were synthesized using a thematic narrative approach. Articles were grouped into conceptual domains reflecting the aims of the review:

(1) Shared biological pathways linking metabolic dysfunction to cardiovascular, renal, hepatic, and endocrine systems;
(2) Clinical and diagnostic overlap across organ systems;
(3) Cross-organ biomarkers and mechanistic intersections;
(4) Implications for precision and preventive medicine.

Only peer-reviewed original research, narrative reviews, systematic reviews, and consensus statements relevant to adult human populations were included. Exclusion criteria comprised animal-only studies without translational relevance, pediatric-only studies, case reports, editorials, and articles focused solely on a single organ system without metabolic context.

The search identified 735 records (717 through database searches and 18 through manual citation screening). After removal of 302 duplicate records, 415 records underwent title and abstract screening, of which 324 were excluded. A total of 91 full-text articles were sought for retrieval, and 90 were successfully assessed for eligibility; 73 of these were excluded for reasons including lack of multisystem metabolic relevance, non-eligible article type, or insufficient mechanistic content. An additional 18 full-text articles identified through manual searching were also assessed, of which 15 were excluded due to conceptual irrelevance or overlap with included studies. Ultimately, 20 studies were included in the narrative synthesis (17 from database searches and 3 from manual methods).

**Figure 1:**
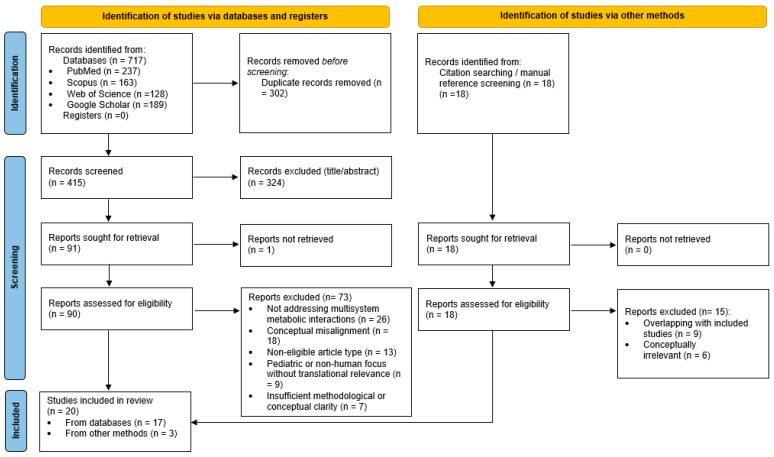
PRISMA 2020 flow diagram illustrating the study selection process for the narrative review.

To enhance transparency of study selection, a PRISMA 2020 flow diagram (Figure 1) is provided, summarizing identification, screening, eligibility assessment, and final inclusion of articles.

The main characteristics of these studies are summarized in Supplementary Table S1.

## 3. Results

The included studies comprised a heterogeneous body of evidence reflecting the multisystem nature of metabolic dysfunction. As summarized in Supplementary Table S1, the literature encompassed narrative and systematic reviews, large observational and population-based cohort studies, randomized controlled trials, and consensus frameworks, predominantly involving adult populations. Across studies, the primary focus was on shared pathophysiological mechanisms, epidemiologic overlap, integrative risk frameworks, biomarkers, and therapeutic effects linking cardiovascular, renal, hepatic, and metabolic systems, supporting a cross-organ, systems-level perspective rather than isolated organ-specific outcomes.

### 3.1. Core Systemic Mechanisms of Metabolic Dysfunction

Metabolic dysfunction represents a unifying, system-wide pathological process through which disturbances in energy handling, lipid trafficking, inflammatory signaling, and redox balance propagate injury across cardiovascular, renal, hepatic, and endocrine systems. Rather than acting as isolated abnormalities, hyperglycemia, insulin resistance, lipotoxicity, oxidative stress, and neurohormonal activation form an interconnected network that drives parallel organ damage and clinical multimorbidity. Accumulating mechanistic and clinical evidence supports the concept that these processes reinforce one another across tissues, establishing a self-sustaining cycle of cardio–renal–hepatic–metabolic deterioration [10].

At the center of this network lies systemic insulin resistance, which disrupts substrate utilization and promotes maladaptive glucose and lipid fluxes across multiple organs. Impaired insulin signaling in skeletal muscle, liver, and adipose tissue leads to reduced glucose uptake, enhanced hepatic gluconeogenesis, and elevated circulating free fatty acids. These alterations promote endothelial dysfunction, vascular inflammation, and atherogenesis, while simultaneously contributing to myocardial remodeling and renal microvascular injury. Chronic hyperglycemia further amplifies oxidative stress through mitochondrial reactive oxygen species generation and downstream pathways that impair endothelial integrity, collectively contributing to progressive declines in estimated glomerular filtration rate and the development of albuminuria [10].

Endothelial dysfunction has emerged as a central and unifying mechanism linking metabolic syndrome to multisystem organ damage. Chronic metabolic stressors, including insulin resistance, dyslipidemia, hypertension, and adipose tissue dysfunction, promote oxidative stress and impair endothelial nitric oxide bioavailability, resulting in reduced vasodilation, heightened vascular inflammation, and a prothrombotic state. Excess reactive oxygen species drive endothelial nitric oxide synthase uncoupling, amplify inflammatory signaling, and upregulate adhesion molecules, thereby facilitating leukocyte recruitment and microvascular injury across vascular beds [11,12]. In parallel, metabolic syndrome is characterized by an imbalance between vasodilatory mediators (e.g., nitric oxide, prostacyclin, endothelium-derived hyperpolarizing factors) and vasoconstrictive pathways (e.g., endothelin-1), further accelerating vascular dysfunction and end-organ injury [12]. In result, these processes position endothelial dysfunction not merely as a downstream consequence of metabolic disease, but as a key upstream driver of cardiovascular, renal, and metabolic complications associated with systemic metabolic dysfunction [11].

Adipose tissue dysfunction constitutes a key upstream driver of these systemic abnormalities. In states of sustained positive energy balance, limited adipocyte expandability results in adiposopathy, characterized by adipocyte hypertrophy, local hypoxia, macrophage infiltration, and dysregulated adipokine secretion. This dysfunctional phenotype promotes chronic low-grade inflammation and excessive free fatty acid release. Subsequent lipid spillover into non-adipose tissues leads to ectopic fat accumulation in the liver, myocardium, pancreas, and renal sinus, exposing these organs to lipotoxic intermediates that impair mitochondrial function, induce endoplasmic reticulum stress, and activate apoptotic and fibrotic pathways [9].

These mechanisms are integrated within the CARDIAL-MS model proposed by Godoy-Matos and colleagues, in which ectopic fat deposition and adipose-derived inflammatory signaling initiate parallel injury across hepatic, cardiovascular, renal, and endocrine systems. In this framework, insulin-resistant adipose tissue drives hepatic de novo lipogenesis and toxic lipid accumulation, leading to mitochondrial dysfunction and altered hepatokine secretion. The steatotic liver thereby acts as a metabolic amplifier, exacerbating systemic insulin resistance, atherogenic dyslipidemia, and inflammatory signaling, and propagating injury to the heart, kidneys, and vasculature through endocrine and paracrine mechanisms [9].

Metabolic dysfunction-associated steatotic liver disease (MASLD) occupies a central position within this multisystem network. Human studies consistently demonstrate that MASLD is associated with increased cardiovascular and renal risk independent of traditional cardiometabolic factors, supporting its classification as a systemic metabolic disorder rather than a liver-restricted condition. Mechanistically, hepatic insulin resistance and lipotoxicity activate pro-inflammatory pathways, including NF-κB signaling, NLRP3 inflammasome activation, and increased secretion of cytokines such as interleukin-6 and tumor necrosis factor-α. These processes drive hepatic fibrogenesis while simultaneously contributing to extrahepatic organ injury, consistent with a “multiple parallel hits” model of metabolic stress [13]. Endocrine dysregulation further modulates MASLD severity and systemic metabolic instability. Alterations in growth hormone–insulin-like growth factor-1 (GH–IGF-1) signaling promote visceral adiposity and impair lipid oxidation, while thyroid hormone abnormalities disrupt hepatic cholesterol metabolism and mitochondrial β-oxidation. Excess glucocorticoid exposure and disturbances in sex hormone signaling further exacerbate adipose dysfunction, inflammation, and fibrotic progression. Together, these endocrine perturbations position MASLD as both a target and an amplifier of hormonal imbalance within multisystem metabolic disease, underscoring endocrine dysregulation as a core upstream mechanism in CRHM pathophysiology [14].

The systemic consequences of hepatic metabolic dysfunction are particularly evident in the kidney. Large observational cohorts and meta-analyses demonstrate that metabolic dysfunction-associated fatty liver disease is independently associated with increased prevalence and incidence of chronic kidney disease. Also, disease severity modifies renal risk: advanced hepatic fibrosis confers substantially stronger associations with CKD than steatosis alone, indicating that progressive liver injury reflects a broader metabolic and inflammatory burden affecting renal microvasculature and tubular function [15,16]. These findings support liver fibrosis as a marker of systemic metabolic toxicity rather than an isolated hepatic outcome.

Oxidative stress and chronic inflammation further integrate metabolic injury across organ systems. Excess nutrient availability, mitochondrial dysfunction, and impaired antioxidant defenses promote sustained redox imbalance, damaging vascular endothelium, accelerating myocardial fibrosis, and sensitizing renal tissue to ischemic and inflammatory injury. In parallel, persistent low-grade inflammation driven by adipose tissue dysfunction, the gut–liver axis, and innate immune activation reinforces insulin resistance and fibrotic remodeling. Neurohormonal overactivation, including the renin–angiotensin–aldosterone system and sympathetic nervous system, creates a feed-forward loop that accelerates cardio–renal–hepatic deterioration [10].

All of these converging pathways demonstrate that metabolic dysfunction operates as a central pathogenic hub linking cardiovascular, renal, hepatic, and endocrine systems through shared mechanisms of insulin resistance, ectopic fat accumulation, inflammation, oxidative stress, endothelial dysfunction, and hormonal imbalance. The severity-dependent associations observed across organ systems indicate that progressive metabolic injury, rather than isolated metabolic abnormalities, drives multisystem disease trajectories. This integrated mechanistic framework provides the biological foundation for CRHM and explains why cardiometabolic diseases commonly coexist, evolve in parallel, and respond to therapies targeting upstream metabolic dysfunction rather than single-organ endpoints [9,10,11].

**Figure 2:**
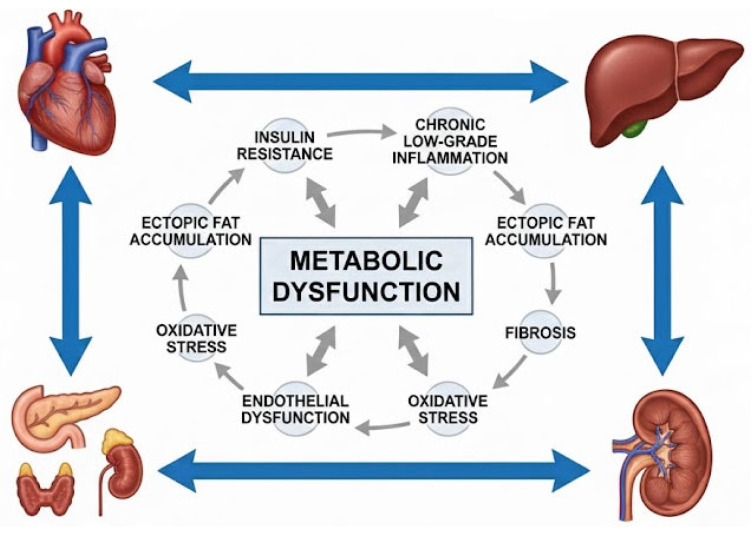
Metabolic dysfunction as a central systemic mechanism linking cardiovascular, renal, hepatic, and endocrine systems. Metabolic dysfunction acts as a central pathogenic hub connecting major organ systems through shared mechanisms including insulin resistance, chronic low-grade inflammation, ectopic fat accumulation, oxidative stress, endothelial dysfunction, fibrosis, and hormonal dysregulation. Bidirectional interactions between the heart, liver, kidney, and endocrine system highlight the systemic nature of cardiometabolic disease beyond isolated organ involvement.

The convergence of these mechanisms across organ systems is schematically illustrated in Figure 2.

### 3.2. Clinical Overlap Across Organ Systems

Clinical and population-level evidence consistently demonstrates that cardiometabolic disorders rarely occur in isolation. Obesity, type 2 diabetes, chronic kidney disease, heart failure, and MASLD frequently cluster within individuals, reflecting shared upstream metabolic drivers rather than independent disease processes. This pattern is captured by contemporary CKM staging frameworks, which conceptualize disease progression as a continuum characterized by the parallel accumulation of metabolic, renal, and cardiovascular injury rather than a linear, organ-specific sequence [8]. Within this continuum, early stages are dominated by excess or dysfunctional adiposity and metabolic risk factors, followed by the emergence of renal impairment and subclinical cardiovascular damage, and ultimately overt cardiovascular disease with or without advanced kidney dysfunction. Transitions across CKM stages commonly involve concurrent abnormalities across multiple organ systems, supporting the view that metabolic dysfunction manifests clinically as synchronized, multisystem disease rather than isolated organ failure [8]. This clinical overlap mirrors mechanistic evidence showing that insulin resistance, ectopic fat accumulation, inflammation, and endothelial dysfunction act systemically to drive parallel organ injury.

MASLD represents a central clinical node within this overlapping disease spectrum. Accumulating evidence indicates that MASLD is not merely a hepatic manifestation of metabolic dysfunction but a systemic condition closely linked to cardiovascular and renal outcomes. Across diverse populations, individuals with MASLD exhibit higher risks of cardiovascular disease, heart failure, arrhythmias, and chronic kidney disease, independent of traditional cardiometabolic risk factors. Disease severity is a critical modifier: advanced hepatic fibrosis is consistently associated with substantially greater cardiovascular and renal risk than steatosis alone, suggesting that progressive liver injury reflects a broader systemic metabolic and inflammatory burden [13].

Beyond cardiovascular and renal disease, MASLD is also associated with a wide range of extrahepatic conditions, including malignancies affecting gastrointestinal, endocrine, genitourinary, and reproductive organs. Many of these associations are observed in non-cirrhotic stages, underscoring that systemic metabolic dysfunction, rather than end-stage liver disease, is the primary driver of multisystem risk [13].

The integrated nature of these conditions is further supported by clinical frameworks that describe CKM syndrome as a dynamic, bidirectional process. Metabolic dysfunction, kidney impairment, and cardiovascular disease frequently amplify one another through reciprocal interactions involving insulin resistance, dysmetabolism, albuminuria, and subclinical vascular injury. Risk prediction approaches that integrate metabolic variables with measures of kidney function and albuminuria outperform traditional organ-specific models, highlighting the value of unified assessment in capturing real-world multimorbidity [17].

Therapeutic evidence provides additional support for this integrated clinical perspective. Interventions targeting metabolic dysfunction have consistently demonstrated coordinated benefits across cardiovascular and renal outcomes, often occurring in parallel rather than sequentially. Treatments initially developed for glycemic control have been shown to reduce cardiovascular events, heart failure risk, and kidney disease progression, supporting the concept that shared metabolic pathways underlie clinical events across these systems [18,19,20]. These benefits extend to individuals without advanced disease, indicating that metabolic interventions can modify early, overlapping disease trajectories.

Importantly, pharmacologic strategies traditionally employed for cardiovascular risk reduction also appear to exert beneficial effects on MASLD, further supporting the concept of shared cardiometabolic pathophysiology. Statins, beyond their established role in reducing atherosclerotic cardiovascular risk, demonstrate pleiotropic anti-inflammatory, anti-oxidative, and anti-fibrotic effects and have been shown to improve liver enzymes, hepatic steatosis, and histologic features of steatohepatitis in MASLD, while remaining safe across disease stages. Observational studies and meta-analyses further suggest an inverse association between statin use and MASLD severity, including reduced prevalence of steatohepatitis and fibrosis. Similarly, ezetimibe, particularly in combination with statins, has been associated with improvements in hepatic steatosis and insulin resistance in experimental and clinical settings [21]. All of these findings highlight that cardiovascular therapies may confer dual cardio-hepatic benefits, reinforcing the clinical interconnectedness of cardiovascular disease and MASLD within an integrated cardiometabolic framework.

The multisystem nature of metabolic dysfunction is further illustrated by conditions not traditionally classified as metabolic. Population-based studies show that disorders such as osteoarthritis are associated with an increased risk of subsequent cardiovascular–renal–metabolic multimorbidity, independent of conventional risk factors, suggesting that such conditions may serve as clinical markers of underlying metabolic vulnerability [22].

At the population level, nationally representative data consistently demonstrate frequent coexistence of cardiometabolic and renal disease. A substantial proportion of adults exhibit involvement of two or more systems, and a clinically meaningful subset already presents with concurrent cardiovascular, renal, and metabolic disease. The prevalence of these overlapping phenotypes has increased over time, reinforcing multisystem involvement as a common clinical presentation rather than an exception [23].

Longitudinal evidence further supports the interconnected nature of disease progression. Markers of disease severity in one organ system, such as indices of hepatic fibrosis, have been linked to subclinical injury in others, including early vascular changes and progression of coronary artery calcification, independent of traditional cardiovascular risk factors [24]. These findings suggest that organ-specific severity markers may function as indicators of broader systemic pathology.

Collectively, these observations demonstrate that metabolic dysfunction gives rise to overlapping, stage-dependent disease trajectories across cardiovascular, renal, hepatic, and endocrine systems. The consistent clustering of conditions, the modifying role of disease severity, and the parallel response to metabolic therapies strongly support the CRHM framework, in which multisystem disease represents the dominant clinical expression of metabolic dysfunction. Key epidemiologic, clinical, and therapeutic evidence supporting this paradigm is summarized in Table 1.

### 3.3. Shared Biomarkers and Diagnostic Intersections

Growing attention has focused on biomarkers that capture shared pathophysiological processes across organ systems rather than isolated, organ-specific injury. Traditional inflammatory markers, such as C-reactive protein, interleukin-6, and tumor necrosis factor-α, remain widely used but lack specificity for long-term, multisystem risk stratification. In contrast, emerging biomarkers reflect convergent mechanisms, including immune activation, fibrosis, oxidative stress, and metabolic overload, that underlie parallel cardiovascular, renal, hepatic, and metabolic dysfunction.

Among these, soluble urokinase plasminogen activator receptor (suPAR) has emerged as a stable marker of chronic immune activation and vascular injury, with consistent associations across chronic kidney disease progression, atherosclerosis, coronary artery disease, and broader cardiometabolic risk. By reflecting sustained inflammatory signaling rather than acute-phase responses, suPAR represents a candidate integrative biomarker of early multisystem vulnerability. Similarly, galectin-3 captures overlapping fibrotic and inflammatory pathways linking cardiac remodeling, renal fibrosis, and liver disease severity, while growth differentiation factor-15 (GDF-15) integrates signals of mitochondrial stress, cellular aging, and metabolic overload [25]. These biomarkers exemplify “diagnostic intersections” within the CRHM framework, offering mechanistic insight and potential utility for early risk stratification, although routine clinical implementation remains limited.

Beyond novel biomarkers, routinely available clinical measures already provide meaningful information on multisystem metabolic injury. Estimated glomerular filtration rate and albumin–creatinine ratio function not only as renal markers but also as indicators of systemic vascular stress within the cardiovascular–kidney–metabolic continuum [8]. Composite metabolic indices, including the triglyceride–glucose (TyG) index, TyG-derived obesity indices, and the atherogenic index of plasma, integrate dysglycemia, dyslipidemia, and adiposity to capture cardiometabolic risk across CKM stages. In parallel, blood count-derived inflammatory indices and cardiac biomarkers—such as NT-proBNP, high-sensitivity troponin, and ST2—aid in identifying subclinical cardiovascular injury within broader metabolic phenotypes [8]. These measures demonstrate how shared diagnostic domains can quantify overlapping organ stress without reliance on siloed organ-specific tests.

Post-translational protein modifications further expand the spectrum of integrative biomarkers. Circulating carbamylated albumin has been associated with increased arterial stiffness, greater coronary atherosclerotic burden, and higher cardiovascular and all-cause mortality, even in individuals with no or mild chronic kidney disease. Its correlation with pulse pressure and angiographic severity scores, along with incremental prognostic value beyond established risk models, suggests that protein carbamylation reflects cumulative metabolic and inflammatory injury bridging cardiovascular and renal pathology within CRHM [26]. Markers of oxidative stress also capture early system-level injury. In large population-based cohorts, higher baseline serum peroxiredoxin-4 (Prx4) has been independently associated with incident chronic kidney disease after adjustment for traditional cardiometabolic risk factors and baseline renal function. The results support redox imbalance as a contributor to renal vulnerability and position Prx4 as a candidate biomarker at the intersection of metabolic, vascular, and renal disease [27]. Prospective data further support the utility of integrative metabolic indices across early CKM stages. TyG-based indices, including TyG–body mass index and TyG–waist circumference, have demonstrated robust associations with cardiovascular disease incidence and mortality in nationwide cohorts of adults with CKM stages 0–3, consistently outperforming body mass index alone in risk discrimination analyses. These observations highlight the capacity of composite metabolic indices to capture systemic cardiometabolic risk beyond isolated measures of adiposity or glycemia [28].

The systemic nature of metabolic dysfunction-associated steatotic liver disease is similarly reflected by shared inflammatory and fibrotic biomarkers. Elevated circulating inflammatory markers and fibrosis-based indices correlate with adverse cardiovascular, renal, and oncologic outcomes, underscoring their role as cross-organ risk indicators within CRHM models [11]. Supporting this concept, large quantitative syntheses demonstrate that non-invasive liver fibrosis scores, including FIB-4, NAFLD Fibrosis Score, and APRI, are consistently associated with major adverse cardiovascular events and cardiovascular mortality, independent of traditional risk factors and diabetes status. These findings position liver fibrosis indices not as liver-specific tools, but as accessible markers of systemic metabolic and inflammatory burden [29].

Shared biomarkers within the CRHM framework can be broadly classified into mechanistic integrative markers reflecting upstream pathobiology and clinically accessible composite indices capturing multisystem risk in routine practice (Table 2 and Table 3).

### 3.4. Implications for Precision and Preventive Medicine

Despite strong evidence linking metabolic dysfunction-associated MASLD to adverse cardiovascular, renal, and oncologic outcomes, liver disease severity remains largely absent from contemporary cardiovascular risk prediction algorithms. Consequently, cardiometabolic risk is frequently underestimated in individuals with MASLD, particularly in those with non-cirrhotic fibrosis. These gaps highlight the need for integrated, phenotype-based risk assessment strategies that account for multisystem metabolic injury rather than isolated organ-specific endpoints, in line with the CRHM framework [11].

Therapeutic evidence further supports this paradigm shift. Pharmacologic strategies targeting upstream metabolic dysfunction—including glucagon-like peptide-1 receptor agonists, sodium–glucose cotransporter-2 inhibitors, insulin-sensitizing agents, and emerging dual GIP/GLP-1 receptor agonists—have demonstrated coordinated benefits across hepatic, cardiovascular, and renal outcomes. These pleiotropic effects reinforce metabolic dysfunction as a shared, modifiable driver of multisystem disease and suggest that targeting metabolic pathways can simultaneously reduce risk across multiple organ systems [11,30]. Beyond classical metabolic diagnoses, certain clinical phenotypes may serve as early and readily identifiable markers of cardiometabolic vulnerability. Conditions such as osteoarthritis, traditionally viewed as localized musculoskeletal disorders, have been associated with an increased risk of subsequent cardiovascular–kidney–metabolic multimorbidity independent of conventional risk factors. Recognition of such phenotypes may enable earlier risk stratification and targeted preventive interventions in metabolically susceptible populations [20]. At the population level, the high and rising prevalence of overlapping cardiovascular, renal, and metabolic disease underlines the limitations of traditional organ-specific prevention strategies. Epidemiologic data indicate that multisystem metabolic injury is often present long before overt clinical disease emerges. Integrating metabolic, renal, and cardiovascular markers into unified risk stratification models may therefore facilitate earlier identification of high-risk phenotypes and enhance the effectiveness of prevention strategies aligned with precision medicine principles [22]. Liver-derived markers offer particular promise in this context. Longitudinal studies linking non-invasive fibrosis indices to progression of subclinical atherosclerosis suggest that repeated assessment of scores such as FIB-4 can identify individuals undergoing silent vascular injury despite the absence of overt cardiovascular disease. Incorporating hepatic phenotypic markers into preventive frameworks may thus improve early detection of systemic metabolic risk and support individualized intervention strategies within CRHM-oriented models [23].

A more integrated approach is needed for prevention, in which metabolic, hepatic, renal, and cardiovascular risk are evaluated concurrently. Emphasizing shared upstream mechanisms and clinically accessible phenotypes may enable earlier identification of high-risk individuals and more effective prevention of multisystem cardiometabolic disease.

From a clinical perspective, the CRHM framework supports a shift toward integrated risk stratification strategies that evaluate cardiovascular, renal, hepatic, and metabolic injury concurrently rather than sequentially. In practice, this approach may inform earlier intensification of preventive therapies in individuals with high-risk metabolic phenotypes, such as those with MASLD-related fibrosis or early kidney dysfunction, even in the absence of overt cardiovascular disease. At the guideline level, CRHM-oriented models may complement existing CKM frameworks by incorporating hepatic severity markers and metabolic indices into risk algorithms, thereby improving identification of patients with multisystem vulnerability. Finally, preventive strategies aligned with CRHM emphasize upstream metabolic intervention, prioritizing therapies and lifestyle approaches that modify shared drivers of disease progression across organ systems.

## 4. Discussion

The findings synthesized in this review collectively challenge the long-standing paradigm of organ-centered disease classification in cardiometabolic medicine. Traditional frameworks that conceptualize cardiovascular, renal, hepatic, and metabolic disorders as discrete entities are increasingly unable to account for the clinical reality of multimorbidity, bidirectional disease progression, and shared risk trajectories observed across populations. Instead, converging evidence supports metabolic dysfunction as a unifying pathological substrate, with organ-specific manifestations representing different expressions of a common systemic process rather than independent diseases.

Contemporary integrative perspectives emphasize that organs such as the heart, liver, kidney, and adipose tissue function not only as passive targets of metabolic injury but also as active regulators of systemic energy homeostasis. In particular, the heart has emerged as a metabolically dynamic organ capable of influencing whole-body metabolism through substrate utilization, mitochondrial signaling, and cardiokine secretion. These cardiac-derived signals modulate hepatic lipid handling, adipose tissue inflammation, renal hemodynamics, and endocrine pathways, reinforcing the concept of continuous interorgan metabolic cross talk rather than unidirectional injury pathways [31]. Within this framework, metabolic stressors such as insulin resistance, ectopic lipid accumulation, and oxidative imbalance propagate dysfunction across tissues simultaneously, challenging the logic of siloed disease definitions.

This systems-based view aligns with recent narrative syntheses highlighting that cardiovascular–renal–hepatic–metabolic syndromes are driven by tightly interconnected upstream processes, including chronic low-grade inflammation, neurohormonal activation, dysregulated lipid flux, and impaired insulin signaling [32]. From this perspective, fragmented, specialty-based management approaches risk obscuring shared disease drivers and may inadvertently delay recognition of multisystem risk. A metabolic-centric framework, by contrast, provides a conceptual structure capable of accommodating the complexity, overlap, and progression of cardiometabolic disease as it is observed in clinical practice.

The relevance of this shift is particularly evident in heart failure, especially phenotypes with preserved ejection fraction, which are increasingly recognized as systemic metabolic syndromes rather than isolated cardiac disorders. Recent integrative analyses demonstrate that heart failure frequently arises in the context of reciprocal dysfunction across the heart, kidney, liver, and endocrine systems, shaped by shared metabolic and inflammatory stressors rather than primary myocardial pathology alone [33].

Beyond conceptual models, anatomical and imaging data provide further support for metabolic dysfunction as a shared substrate linking organ systems. Advances in multimodality imaging have revealed that visceral and ectopic fat depots—including epicardial, hepatic, pancreatic, and renal sinus fat—are closely associated with parallel cardiovascular, renal, and endocrine injury, independent of body mass index [34]. These depots serve not merely as markers of adiposity but as metabolically active tissues that contribute to local inflammation, fibrosis, and microvascular dysfunction. Their distribution and activity offer a tangible anatomical correlate to the systemic metabolic stress that underlies CRHM phenotypes.

The clinical implications of adopting a metabolic-centric perspective are substantial. Rather than viewing cardiovascular disease, chronic kidney disease, or liver disease as downstream complications that arise sequentially, the CRHM framework reframes these conditions as co-evolving manifestations of a shared metabolic milieu. This reframing helps explain why traditional risk prediction tools, which are often designed around single-organ outcomes, frequently underestimate risk in individuals with overlapping metabolic dysfunction, such as those with MASLD or early renal impairment. Population-based data increasingly demonstrate that metabolic risk accumulates well before overt organ-specific disease becomes clinically apparent. Large prospective cohorts show that composite metabolic indices can capture this early risk more effectively than isolated measures of glycemia, adiposity, or lipid levels. In this context, TyG–based indices have emerged as practical, quantitative markers that integrate dysglycemia, dyslipidemia, and insulin resistance. In a nationwide prospective cohort spanning CKM stages 0–3, higher TyG index values and TyG-derived indices were consistently associated with increased risk of major adverse cardiovascular events and incident chronic kidney disease, with meaningful improvements in risk discrimination when incorporated into baseline models [35]. These outcomes illustrate how metabolic dysfunction can be operationalized as a measurable, system-level risk phenotype rather than an abstract construct. Taken together, these data support a fundamental reorientation of cardiometabolic medicine away from organ-based hierarchies and toward integrated metabolic phenotyping. Such a shift does not negate the importance of organ-specific diagnostics or therapies but rather situates them within a broader systems framework that acknowledges shared drivers, parallel trajectories, and reciprocal amplification across tissues. From a research perspective, this approach encourages study designs that prioritize cross-organ outcomes, longitudinal metabolic profiling, and integrative biomarkers. From a clinical standpoint, it provides a more coherent explanation for the clustering of disease and offers a rationale for earlier, more holistic intervention.

Importantly, the move toward metabolic-centric thinking also has implications for how disease progression is conceptualized and communicated. Rather than framing cardiometabolic disease as a linear sequence—obesity leading to diabetes, followed by cardiovascular or renal complications—the CRHM model emphasizes convergence and simultaneity. Patients frequently enter clinical care at different points along this continuum, often with subclinical involvement of multiple systems. Recognizing this pattern may help clinicians identify high-risk individuals earlier and avoid the false reassurance that can arise when individual organ-specific metrics appear “within range.” In this context, metabolic dysfunction should be viewed less as a background risk factor and more as the central organizing principle of cardiometabolic disease. The accumulating evidence reviewed here suggests that such a reframing better aligns with observed epidemiology, clinical trajectories, and therapeutic responses. Integrated metabolic models are therefore not merely conceptual constructs but practical tools for understanding and addressing the growing burden of multisystem cardiometabolic disease.

Ultimately, replacing organ-based thinking with a metabolic-centric framework represents a necessary evolution in both research and clinical practice. As cardiometabolic multimorbidity becomes the norm rather than the exception, frameworks such as CRHM provide a more accurate, biologically grounded lens through which disease can be studied, prevented, and treated. Future work will need to refine these models, validate integrated risk tools, and determine how best to translate metabolic phenotyping into routine clinical decision-making. Nonetheless, the evidence to date strongly supports metabolic dysfunction as the common denominator linking cardiovascular, renal, hepatic, and endocrine disease across the lifespan.

## 5. Conclusions

This review adopts a metabolic-centric perspective to revisit the overlap between cardiovascular, renal, hepatic, and endocrine disorders. Rather than treating these conditions as independent or sequential entities, the CRHM framework emphasizes metabolic dysfunction as a shared context in which multisystem disease emerges and evolves. This shift moves beyond traditional organ-based thinking and reflects the growing recognition that cardiometabolic multimorbidity is the dominant clinical pattern in contemporary practice.

Viewing metabolic dysfunction as a central organizing principle has important implications for how risk is conceptualized and managed. Organ-specific approaches, while essential for diagnosis and treatment, may overlook broader systemic vulnerability, particularly in individuals with early or subclinical disease across multiple systems. Integrated frameworks such as CRHM provide a structure for considering liver, kidney, cardiovascular, and metabolic health in parallel, offering a more coherent approach to risk assessment and prevention.

From a clinical standpoint, this perspective supports greater attention to metabolic phenotypes, disease severity markers, and cross-organ indicators that may signal increased vulnerability before advanced disease becomes apparent. From a research standpoint, it encourages the development and validation of integrated risk tools, longitudinal phenotyping strategies, and study designs that reflect multisystem disease trajectories rather than isolated outcomes.

As the prevalence of cardiometabolic multimorbidity continues to rise, frameworks that center on metabolic dysfunction may help bridge gaps between specialties and support more individualized, prevention-oriented care. While further work is needed to translate these concepts into routine clinical workflows, adopting a metabolic-centric view represents a necessary step toward more integrated and forward-looking management of complex cardiometabolic disease.

## 6. Limitations and Future Directions

This review has several limitations. First, its narrative design, while suitable for conceptual integration across heterogeneous evidence, lacks the quantitative rigor of a systematic review or meta-analysis. Despite the structured search strategy and PRISMA-guided selection, the synthesis remains interpretive, and causal inferences regarding shared metabolic mechanisms across organ systems cannot be firmly established. Second, much of the evidence underpinning the CRHM framework derives from observational studies, post hoc analyses, and heterogeneous meta-analyses. Although these consistently demonstrate parallel multisystem involvement, residual confounding and uncertainty regarding the temporal sequencing of organ dysfunction persist. It therefore remains unclear whether specific organ manifestations represent primary drivers, downstream consequences, or markers of cumulative metabolic burden. Finally, the reviewed literature predominantly reflects adult populations from high- and middle-income settings, with limited representation of pediatric populations, diverse ethnic groups, and low-resource environments. Future research should prioritize prospective validation of integrated metabolic biomarkers, development of CRHM-oriented risk prediction tools, and clinical trials specifically designed to assess predefined multisystem outcomes. These efforts will be critical for translating metabolic-centric frameworks into routine clinical practice and precision prevention strategies.

## Figures and Tables

**Table 1:** Clinical and Epidemiologic Evidence Supporting Overlapping Cardiovascular–Renal–Hepatic–Metabolic Disease Trajectories.

Index Condition	Overlapping Systems	Key Quantitative Evidence	Severity/Stage Effect
CKM syndrome	CV + renal + metabolic	~25% of adults had ≥1 CKM condition; ~8% exhibited concurrent CV–renal–metabolic multimorbidity [8,23]	Cardiovascular events and mortality increased progressively from CKM stage 0 to 4 [8,17]
MASLD	Hepatic + CV + renal	Meta-analyses (>5 million individuals) show increased risk of cardiovascular events and heart failure (HR ≈ 1.4–1.5) [11]	Risk rose stepwise with fibrosis severity; HR > 2.0 in advanced fibrosis/cirrhosis [11]
MASLD with fibrosis (FIB-4)	Hepatic + CV	Higher FIB-4 associated with progression of coronary artery calcification independent of traditional risk factors [24]	Associations strongest in intermediate–high fibrosis categories [24]
Type 2 diabetes	Metabolic + CV + renal	GLP-1RAs and SGLT2 inhibitors reduced major CV events, heart failure, and kidney disease progression in RCT meta-analyses [18,19]	Benefits consistent across baseline CV risk and kidney function strata [18,19]
Obesity (without diabetes)	Metabolic + CV	Targeting obesity reduced major adverse CV events and all-cause mortality in outcome trials [20]	Effects consistent across BMI and prediabetes subgroups [20]
Osteoarthritis	Musculoskeletal + CV + renal + metabolic	Higher incidence of new-onset CKM multimorbidity compared with controls in population cohorts [22]	Clear dose–response across osteoarthritis severity strata [22]
Population-level cardiometabolic multimorbidity	CV + renal + metabolic	Dual and triple-system involvement increased over time in nationally representative cohorts [23]	Multimorbidity markedly higher in older adults (≥65 years) [23]

Abbreviations: CV, cardiovascular; CKM, cardiovascular–kidney–metabolic; FIB-4, fibrosis-4 index; HR, hazard ratio; MASLD, metabolic dysfunction-associated steatotic liver disease; SGLT2, sodium–glucose cotransporter-2; GLP-1RA, glucagon-like peptide-1 receptor agonist.

**Table 2:** Mechanistic and Emerging Integrative Biomarkers Reflecting Multisystem CRHM Dysfunction.

Biomarker	Primary Pathophysiological Domain	Major Organ Systems Involved	Clinical and Prognostic Associations
suPAR	Chronic immune activation; endothelial dysfunction	Renal · Cardiovascular · Metabolic	Elevated suPAR is associated with CKD progression, atherosclerosis, coronary artery disease, and systemic inflammation, reflecting sustained immune activation rather than an acute-phase response.
Galectin-3	Fibrosis; inflammation	Cardiovascular · Renal· Hepatic	Increased galectin-3 concentrations are linked to cardiac remodeling, renal fibrosis, hepatic fibrogenesis, and higher mortality in heart failure and chronic liver disease.
GDF-15	Mitochondrial stress; cellular aging	Cardiovascular · Renal · Metabolic	GDF-15 is associated with cardiometabolic stress, cardiovascular aging, and adverse cardiovascular outcomes, integrating mitochondrial dysfunction across multiple organ systems.
Prx4	Oxidative stress; redox imbalance	Renal · Cardiovascular · Metabolic	Elevated circulating Prx4 independently predicts incident CKD and is associated with cardiometabolic risk and all-cause mortality, indicating systemic oxidative stress preceding overt organ damage.
C-Alb	Protein carbamylation; inflammation; vascular stiffness	Cardiovascular · Renal · Metabolic	Higher C-Alb levels correlate with arterial stiffness, coronary artery disease severity, systemic inflammation, and independently predict all-cause and cardiovascular mortality, including in individuals without advanced CKD.
Leptin	Adipokine dysregulation	Metabolic · Cardiovascular · Renal	Elevated leptin levels are associated with obesity-related cardiometabolic risk, endothelial dysfunction, reduced eGFR, and albuminuria, linking dysfunctional adiposity to multi-organ injury.

Abbreviations: CKD, chronic kidney disease; C-Alb, carbamylated albumin; GDF-15, growth differentiation factor-15; Prx4, peroxiredoxin-4; suPAR, soluble urokinase plasminogen activator receptor.

**Table 3:** Clinically Accessible Composite Indices and Routine Markers Capturing Multisystem CRHM Risk.

Biomarker/Index	Primary Pathophysiological Domain	Major Organ Systems Involved	Clinical and Prognostic Associations
FIB-4 index	Fibrosis; chronic inflammation	Hepatic · Cardiovascular · Renal	Elevated FIB-4 is associated with increased cardiovascular event risk, demonstrating a dose–response relationship that persists irrespective of NAFLD/MASLD status.
NFS	Fibrosis; metabolic stress	Hepatic · Cardiovascular · Renal	Higher NFS is associated with substantially increased cardiovascular risk, independent of overt NAFLD, supporting fibrosis burden as a systemic risk marker.
APRI	Fibrosis; systemic inflammation	Hepatic · Cardiovascular	Elevated APRI is associated with increased cardiovascular event risk, reflecting advanced metabolic–hepatic injury with cardiovascular implications.
Liver fibrosis severity (imaging-based or composite scores)	Fibrosis; insulin resistance; chronic inflammation	Hepatic · Cardiovascular · Renal · Metabolic	Progressive MASLD-related fibrosis is independently associated with major adverse cardiovascular events, heart failure, atrial fibrillation, CKD progression, and extrahepatic complications.
TyG index and modified TyG indices (TyG-BMI, TyG-WC, TyG-WHtR)	Insulin resistance; metabolic overload	Metabolic · Cardiovascular · Renal	Higher TyG-based indices are associated with incident cardiovascular disease and more advanced CKM stages; modified indices outperform TyG alone in cardiometabolic risk prediction.
Novel adiposity indices (WWI, ABSI, WHtR, Conicity index)	Visceral adiposity; adipose tissue dysfunction	Cardiovascular · Renal · Metabolic	These indices show strong linear associations with all-cause and cardiovascular mortality across CKM stages 0–3, with WWI showing the strongest predictive value, outperforming BMI.
Serum uric acid	Oxidative stress; adverse metabolic milieu	Cardiovascular · Renal · Metabolic · Hepatic	Hyperuricemia is associated with atherosclerosis, CKD, type 2 diabetes, obesity, MASLD, and increased cardiovascular morbidity and mortality.
SII, SIRI	Chronic low-grade systemic inflammation	Cardiovascular · Renal · Metabolic	Elevated indices are associated with advanced CKM stages and increased all-cause and cardiovascular mortality, reflecting persistent multisystem inflammation.
eGFR and UACR	Microvascular dysfunction; renal–vascular interface	Renal · Cardiovascular · Metabolic	Reduced eGFR and increased albuminuria serve as integrative markers of systemic vascular injury and are central to CKM staging and risk stratification.

Abbreviations: ABSI, A Body Shape Index; APRI, aspartate aminotransferase-to-platelet ratio index; BMI, body mass index; CKD, chronic kidney disease; CKM, cardiovascular–kidney–metabolic; eGFR, estimated glomerular filtration rate; FIB-4, fibrosis-4 index; MASLD, metabolic dysfunction-associated steatotic liver disease; NAFLD, non-alcoholic fatty liver disease; NFS, NAFLD Fibrosis Score; SII, systemic immune-inflammation index; SIRI, systemic inflammation response index; TyG, triglyceride–glucose index; TyG-BMI, TyG combined with body mass index; TyG-WC, TyG combined with waist circumference; TyG-WHtR, TyG combined with waist-to-height ratio; UACR, urine albumin-to-creatinine ratio; WHtR, waist-to-height ratio; WWI, weight-adjusted waist index.

## Data Availability

No new data were created or analyzed in this study. Data sharing is not applicable to this article.

## Supplementary Materials

The following supporting information can be downloaded at: https://www.mdpi.com/article/10.3390/life16010172/s1, Table S1. Characteristics of Included Studies.

## Abbreviations

The following abbreviations are used in this manuscript:

ASCVD	Atherosclerotic Cardiovascular Disease
BMI	Body mass index
CKD	Chronic Kidney Disease
CKM	Cardiovascular–Kidney–Metabolic
CRH	Cardio-Renal–Hepatic
CRHM	Cardiovascular–Renal–Hepatic–Metabolic
CRP	C-reactive protein
CRS	Cardio-Renal Syndrome
eGFR	Estimated glomerular filtration rate
FIB-4	Fibrosis-4 index
MASH	Metabolic Dysfunction-Associated Steatohepatitis
MASLD	Metabolic Dysfunction-Associated Steatotic Liver Disease
SII	Systemic immune-inflammation index
SIRI	Systemic inflammation response index
TyG	Triglyceride–glucose
T2D	Type 2 Diabetes Mellitus
UACR	Urine albumin-to-creatinine ratio
WHtR	Waist-to-height ratio
WWI	Weight-adjusted waist index
