# Supplementary material for: Metabolic Dysfunction at the Core: Revisiting the Overlap of Cardiovascular, Renal, Hepatic, and Endocrine Disorders
Source: Life (Basel). 2026 Jan 20;16(1):172. doi: 10.3390/life16010172 (PMC12842696; doi:10.3390/life16010172)
Supplement: Supplementary file 1 [file life-16-00172-s001.zip › Supplementary_Table_S1_Characteristics_of_Incuded_Studies.pdf]

**Table S1. Characteristics of Included Studies**

| First Author (Year) | Study Design | Population / Condition | Systems Involved | Primary Focus | Key Findings |
|---|---|---|---|---|---|
| Mutruc et al. (2025) [8] | Narrative review | CKM-related cardiometabolic multimorbidity | Cardiovascular; renal; metabolic | Pathophysiology, epidemiology, and staging of CKM syndrome | Identifies shared mechanisms linking cardiovascular disease, chronic kidney disease, obesity, and type 2 diabetes within a unified CKM framework. |
| Godoy-Matos et al. (2025) [9] | Narrative review | Metabolic syndrome, MASLD, cardiometabolic disease | Cardiovascular; renal; hepatic; pancreatic; adipose; vascular | CARDIAL-MS multisystem metabolic framework | Emphasizes adipose dysfunction, ectopic fat, and hepatokine imbalance as drivers of multisystem metabolic injury; proposes a four-stage CARDIAL-MS model. |
| Marassi & Fadini (2023) [10] | Narrative review | Adults with CKM syndrome | Cardiovascular; renal; metabolic; vascular | CKM staging and integrated risk framework | Demonstrates strong epidemiologic clustering of diabetes, cardiovascular disease, and CKD, with bidirectional risk amplification. |
| Targher et al. (2024) [13] | Narrative review | Adults with MASLD/MASH | Hepatic; cardiovascular; renal; metabolic; oncologic | Systemic complications of MASLD | MASLD independently associated with cardiovascular events, CKD, and extrahepatic cancers; risk increased with fibrosis severity. |
| Hutchison et al. (2023) [14] | Narrative review | Adults with MASLD/MASH | Hepatic; endocrine; metabolic | Endocrine regulation of MASLD | Hormonal axes modulate hepatic steatosis, fibrosis, and systemic insulin resistance, positioning MASLD as an endocrine–metabolic disorder. |
| Agustanti et al. (2023) [15] | Systematic review and meta-analysis | Adults with MAFLD | Metabolic; renal | MAFLD–CKD association | MAFLD associated with higher prevalent and incident CKD; renal risk increased with advanced hepatic fibrosis. |
| Zhou et al. (2023) [16] | Systematic review and meta-analysis | Adults with MAFLD | Hepatic; renal; metabolic | MAFLD and CKD risk | Fibrosis-based MAFLD phenotypes conferred greater CKD risk than steatosis alone. |
| Massy & Drueke (2025) [17] | Narrative review | CKM syndrome framework | Cardiovascular; renal; metabolic; vascular | Integrated CKM staging and risk prediction | Shows that inclusion of eGFR and albuminuria improves adverse outcome prediction across CKM stages. |
| Sattar et al. (2021) [18] | Systematic review and meta-analysis of RCTs | Adults with type 2 diabetes | Cardiovascular; renal; metabolic; vascular | Cardiovascular and renal outcomes of GLP-1 receptor agonists | GLP-1 receptor agonists reduced major cardiovascular events, mortality, and kidney disease progression, indicating parallel cardio–renal benefit. |
| McGuire et al. (2021) [19] | Systematic review and meta-analysis of RCTs | Adults with type 2 diabetes | Cardiovascular; renal; metabolic | Cardio–renal effects of SGLT2 inhibitors | SGLT2 inhibitors reduced heart failure hospitalization and slowed CKD progression, independent of glycemic control. |
| Lincoff et al. (2023) [20] | Randomized controlled trial | Adults with obesity, no diabetes | Cardiovascular; renal; metabolic | Cardiovascular outcomes of semaglutide | Semaglutide reduced major cardiovascular events and mortality, with favorable renal signals. |

| | | | | | |
|---|---|---|---|---|---|
| Zou et al. (2025) [22] | Population-based prospective cohort | Adults with osteoarthritis | Cardiovascular; renal; metabolic | Osteoarthritis and CKM multimorbidity | Osteoarthritis associated with higher risk of dual and triple CKM multimorbidity and accelerated disease progression. |
| Ostrominski et al. (2023) [23] | Serial cross-sectional population study | U.S. adults (NHANES 1999–2020) | Cardiovascular; renal; metabolic | Population burden of multimorbidity | Over one quarter of adults had ≥1 cardiometabolic or renal condition; ~8% had triple-system involvement. |
| Lee (2025) [24] | Longitudinal cohort study | Adults with MASLD | Hepatic; cardiovascular; metabolic | Liver fibrosis and subclinical atherosclerosis | Time-updated FIB-4 independently predicted progression of coronary artery calcification. |
| Nzobokela et al. (2025) [25] | Narrative review | CRHM-related disorders | Cardiovascular; renal; hepatic; metabolic | Shared mechanisms and biomarkers | Identifies inflammation, oxidative stress, and insulin resistance as shared drivers; highlights suPAR, galectin-3, and GDF-15 as integrative biomarkers. |
| Daschner et al. (2025) [26] | Observational cohort study | Adults undergoing coronary angiography | Cardiovascular; renal | Protein carbamylation and vascular risk | Carbamylated albumin associated with arterial stiffness and increased cardiovascular and all-cause mortality. |
| Geertsema et al. (2024) [27] | Prospective population-based cohort | General population (PREVEND) | Renal; cardiovascular | Oxidative stress and CKD risk | Elevated serum peroxiredoxin-4 independently predicted incident CKD. |
| Zhou et al. (2025) [28] | Prospective cohort study | Adults with CKM stages 0–3 | Cardiovascular; renal; metabolic | Adiposity-based metabolic risk indices | TyG-derived indices outperformed BMI in predicting cardiovascular and all-cause mortality. |
| Zhang et al. (2025) [29] | Systematic review and meta-analysis | Adults with and without NAFLD | Cardiovascular; hepatic; metabolic | Prognostic value of fibrosis scores | FIB-4 and NAFLD Fibrosis Score predicted cardiovascular events and mortality independent of NAFLD status. |
| Theodorakis & Nikolaou (2025) [30] | Narrative review | Adults with cardiometabolic disease across CKM and CRHM spectra | Cardiovascular; renal; hepatic; metabolic | Integrated management of CRHM syndrome and role of metabolic therapies | Extends CKM to a CRHM framework incorporating MASLD and highlights coordinated cardio-renal-hepatic benefits of contemporary metabolic therapies. |
